# Fire from Rubber Goods

**Published:** 1881-09

**Authors:** 


### FIRE FROM RUBBER GOODS.

An interesting illustration of the danger attending the manufacture of some kinds of rubber goods was shown in the origin of the recent fire which occured in the Ætna Rubber Mills at Jamaica Plains. The cement which fastens the seams of rubber coats is largely made of naphtha. The mere act of lifting a piece of rubber cloth from a pile of half-a-dozen similar ones, cut for garments, developed so much electricity that a spark was observed to escape. It came in contact with the naphtha cement, or with gases arising from it, and instantly the whole room was in a blaze.

Fortunately the fire was extinguished without destroying the mill, the loss being only about a thousand dollars.

It is not known that anything can be done to prevent the occurrence of another accident of precisely the same kind, whenever all the atmospheric conditions are favorable. One would suppose, however, that a certain degree of dampness would remove all danger from that source.—*Commercial Bulletin.*

